# Arbitration of Approach-Avoidance Conflict by Ventral Hippocampus

**DOI:** 10.3389/fnins.2020.615337

**Published:** 2020-12-17

**Authors:** Kathleen G. Bryant, Jacqueline M. Barker

**Affiliations:** Department of Pharmacology and Physiology, Drexel University, Philadelphia, PA, United States

**Keywords:** ventral hippocampus (vHPC), conflict, arbitration, fear, reward

## Abstract

When environmental cues or stimuli that represent both rewarding and aversive outcomes are presented, complex computations must be made in order to determine whether approach or avoidance is the better behavioral strategy. In many neuropsychiatric illnesses these computations can be skewed. In some instances, circumstances that may normally warrant avoidance instead promote approach, thus producing compulsive-like behavioral strategies that are inflexible in response to new or conflicting information. Alternatively, high sensitivity to aversion or low sensitivity to reward can result in the failure to achieve goals and loss of resilience that characterizes depressive disorders. Increases in compulsive-like behavior have been found to be associated with disrupted signaling in regions that regulate response to conflicting stimuli, including the hippocampus. Classic behavioral inhibition theories of hippocampus function in anxiety suggest that the hippocampus blocks aberrant behavior in response to anxiety related cues or stimuli. The hippocampus may act to block approach in the face of conflicting stimuli. Dysregulations of hippocampal function, as may be present in neuropsychiatric disorders, may therefore promote aberrant approach behavior. The ventral hippocampus (vHPC) subregion is key for coordinating this approach/avoidance conflict resolution, likely through its participation with cortico-striatal and mesolimbic circuits. We revisit Gray's behavioral inhibition theory of HPC function, first posited in the 1980s, and interpret in the context of new knowledge on vHPC function gained through modern technology. Taken together with the extant, classical literature on hippocampal function, we propose that these new findings suggest that vHPC circuits balance behavioral response to conflicting stimuli in a manner that is both state- and context-dependent and, further, that disruption of specific vHPC circuits tips the balance in favor of biased approach or avoidance behavioral strategies.

## Introduction

**Figure 1:**
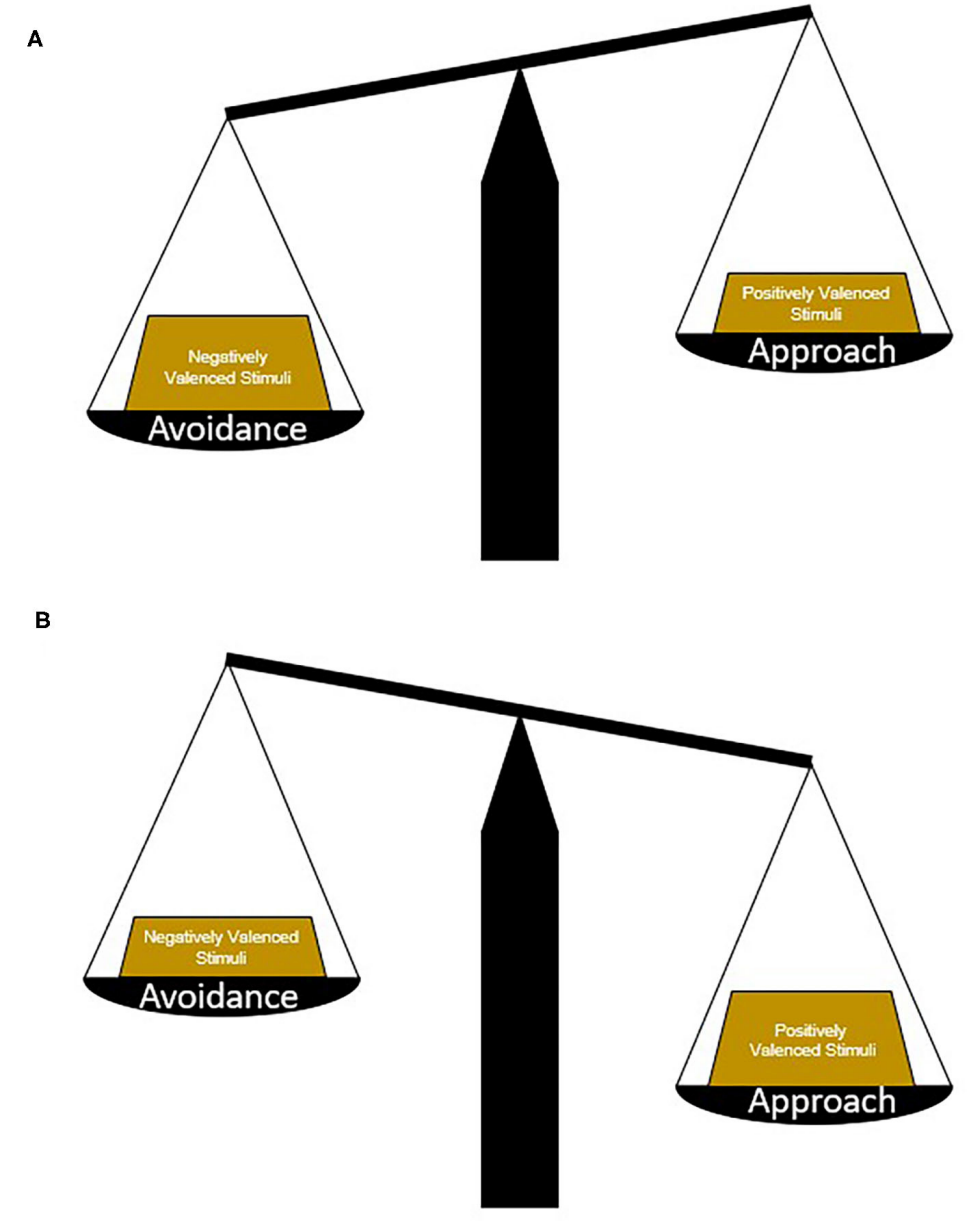
Determining approach or avoidance strategies. Picking an appropriate behavioral strategy in response to conflicting stimuli requires the weighing and balancing of potential risks vs. benefits. In general, avoidance strategies are chosen in circumstances where negatively valenced stimuli hold more weight or the potential risks are greater than the potential benefits **(A)**. In contrast, approach strategies are chosen in circumstances where positively valenced stimuli hold more weight or the potential benefits are greater than the potential risks **(B)**. In neuropsychiatric illnesses and diseases, maladaptive strategies may be chosen because of changes in the weight of different stimuli, a change in the ratio of positively and negatively valenced stimuli, or biases toward certain response patterns.

Environmental cues and contexts that signal positive or desirable outcomes generally promote approach whereas those that signal negative or unpleasant outcomes promote avoidance (Gray, 1982; Elliot, 2006). An approach-avoidance conflict can arise in cases where a cue or context represents both positive and negative outcomes. In such circumstances more complex computations weighing the potential risks and benefits of approaching or avoiding are required in order to choose the most appropriate behavioral response (Figure 1). For example, when the potential for reward is high and the risk is small, an approach behavioral response is chosen, while high risk, small reward circumstances yield avoidance. The ability to accurately perceive, balance, and weigh conflicting environmental information may be disrupted in some neuropsychiatric illnesses. This can cause an overreliance either on approach-related behaviors—resulting from increased attribution of salience or value to positive valence stimuli relative to negative valence stimuli – or, conversely, on avoidance-related behaviors when negative valence stimuli dominate behavioral response. Imbalance in approach-avoidance behavior can lead to and perpetuate maladaptive behavioral responses that include uncontrolled reward seeking (e.g., compulsivity) or loss of normal goal-directed behavior in the face of effort or mild negative consequences.

Findings from clinical and preclinical literature implicate the hippocampus (HPC) in arbitrating conflicting stimuli (Pennartz et al., 2011; Ito and Lee, 2016). Based on data showing qualitatively similar effects of anxiolytic drugs and HPC lesions on approach-avoidance conflict (Klüver and Bucy, 1937; Gray, 1977; Rickels, 1978), it was postulated by Gray in the 1980s that the HPC acts as a comparator by comparing conflicting stimuli and, further, that as a central component of a septo-hippocampal behavioral inhibition system that the HPC disproportionately weights negatively valenced stimuli (Gray, 1982; McNaughton and Gray, 2000). Thus, the HPC is postulated to block ongoing behavior when there is a mismatch between stimuli and predicted outcomes, allowing for increased attention to the environment. When the HPC is ablated there is no longer a system in place to give enough weight to negatively valued stimuli and thus animals exhibit more approach-related behaviors. This theory of HPC function by Gray et al. was discounted because of data that showed the HPC primarily as a structure involved in spatial navigation and memory consolidation (O'Keefe and Nadel, 1978; Cohen and Eichenbaum, 1993), until future work investigated functional heterogeneity of the HPC (McNaughton, 1997; McNaughton and Gray, 2000; Davidson and Jarrard, 2004).

It is now known that the HPC can be anatomically and functionally separated into dorsal and ventral subregions (posterior and anterior, respectively, in humans and non-human primates) (Fanselow and Dong, 2010; Bach et al., 2014) and the function of these discrete subregions is generally conserved across species (Ito and Lee, 2016). It is further postulated based on molecular and morphological signatures across the dorsal-ventral axis that a third intermediate subregion of the HPC should be considered when defining HPC subregions (Fanselow and Dong, 2010; Lothmann et al., 2020).

Hippocampus functions in memory formation and spatial navigation have been largely attributed to the dorsal HPC, as selective inactivation or ablation of this dorsal region leads to deficits in these areas (Frankland et al., 1998). The ventral hippocampus (vHPC), however, regulates emotional affect and memory, such that vHPC inactivation or ablation leads to robust changes in behavior that cannot be attributed solely to spatial learning or memory recall, meaning that vHPC manipulation can impact behavior without affecting the ability to recall or recognize environmental cues and contexts (Kjelstrup et al., 2002; Fanselow and Dong, 2010). These more recent vHPC findings have led to a surge of scientific interest in this subregion and its projections as they relate to anxiety, fear, and reward seeking. Furthermore, technical advances in projection-specific manipulations has allowed for a greater understanding of how distinct ventral Cornu Ammonis (vCA) and subicular subfields within vHPC contribute to emotional memory.

**Figure 2:**
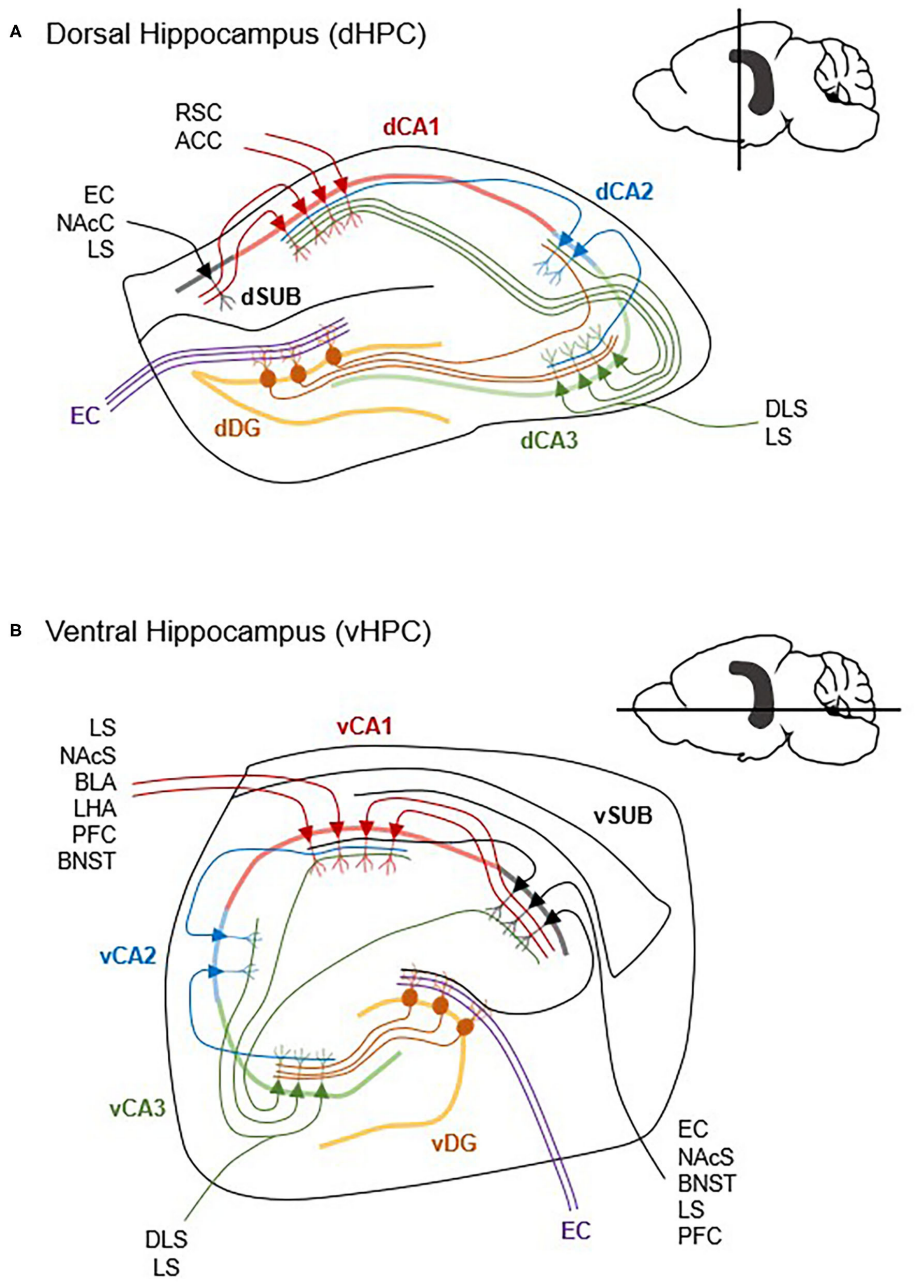
Differences in dorsal and ventral hippocampal circuitry. The hippocampus contains extensive within-region micro circuitry between the dentate gyrus (DG), Cornu Ammonis 1 (CA1), CA2, CA3, and subiculum (SUB) subregions. Generally, input from the entorhinal cortex (EC) (inputs are simplified here) is received by the DG, which projects via mossy fibers to CA3, and then to CA1 via the Schaffer collaterals. Though the dHPC **(A)** and vHPC **(B)** share the same general micro circuitry, vCA1 and vSUB have more reciprocal connections and project to more cortical and subcortical structures than dCA1 and dSUB. ACC, anterior cingulate cortex; BLA, basolateral amygdala; BNST, bed nucleus of the stria terminalis; DLS, dorsolateral striatum; LHA, lateral hypothalamus; LS, lateral septum; NAcC, nucleus accumbens core; NAcS, nucleus accumbens shell; PFC, prefrontal cortex; RSC, retrosplenial cortex (Cenquizca and Swanson, 2007; Witter, 2007; McGinty et al., 2011; Arszovszki et al., 2014; Bienkowski et al., 2018; Besnard et al., 2020; Gergues et al., 2020).

The HPC contains several major, well-defined, and structured lines of pyramidal neurons known as CA1, CA2, and CA3. In canonically defined projections, input from the entorhinal cortex is received by the dentate gyrus, which projects to CA3. Projections from CA3 innervate CA1 and the subiculum. While a subset of entorhinal input is received by CA2 which then projects to CA1, the vast amount of input goes through the dentate gyrus and CA3 (Figure 2). As such, the CA1 and subiculum are the two major outputs from the HPC (Gergues et al., 2020), and thus their connectivity is well-characterized. The CA3 subfield also has substantial extrahippocampal projections (Fanselow and Dong, 2010). Further, many of the papers that specify vCA1 may fail to distinguish from ventral subiculum (vSUB) since they are in such close proximity and this may explain some of the contrasting results of vCA1 manipulation which will require careful parsing in future research. Functionally, the CA and subicular subfields are heterogenous along the dorsal-ventral axis, which is where many of the previously discussed dorsal HPC and vHPC differences arise from. Thus, when possible, vCA and vSUB subregions will be specified herein.

**Figure 3:**
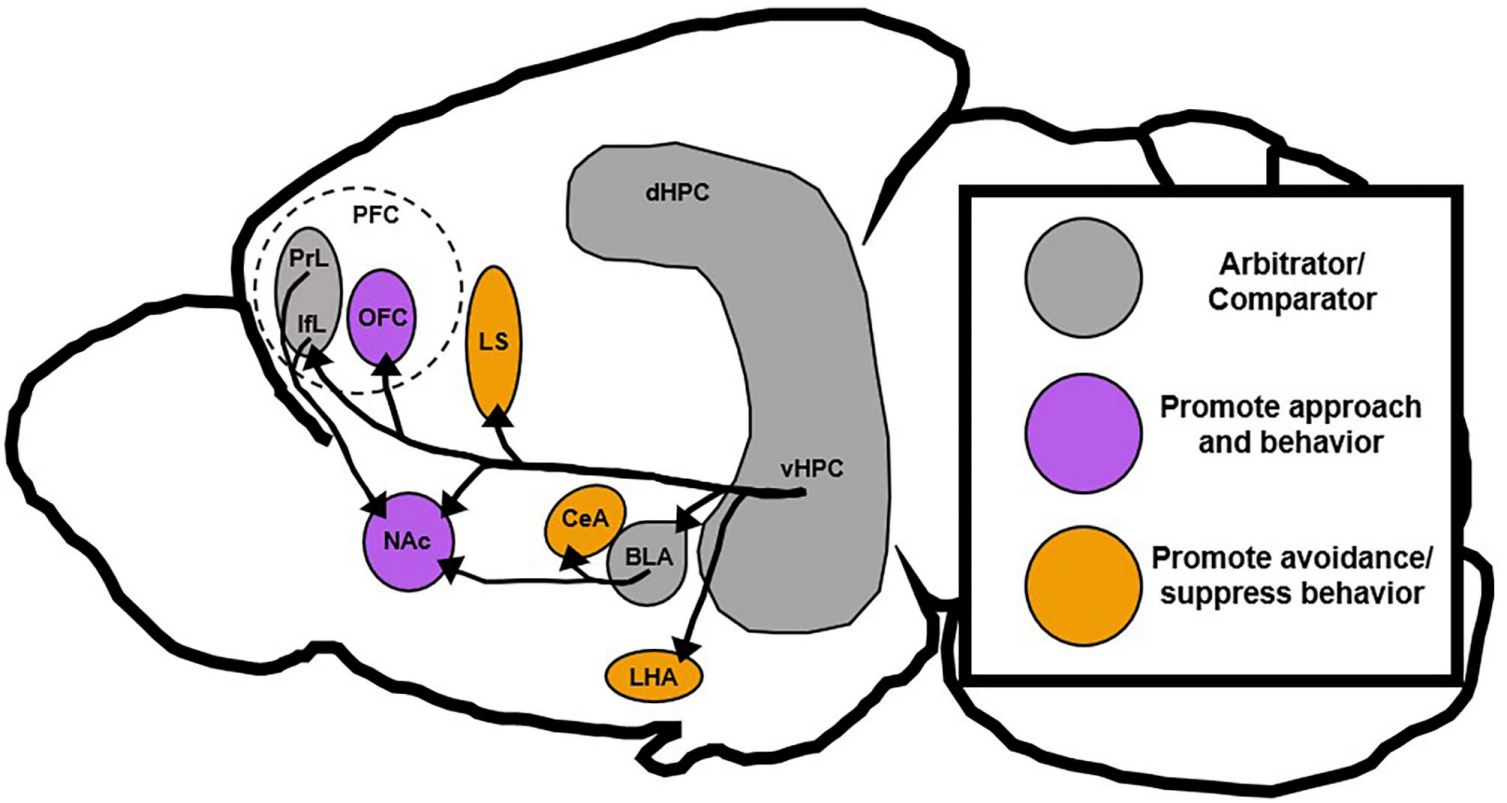
Proposed roles of downstream vHPC targets. The vHPC is posited to be a region that compares conflicting stimuli and signals to inhibit aberrant behavior, but recent data has shown that vHPC can also promote behavioral action. This suggests that vHPC may still act as an arbitrator or comparator between conflicting stimuli, but promote different behavioral responses based on projection target. This review postulates that vHPC projections to orbitofrontal and accumbal regions generally support behavioral action, while those projections to septal and hypothalamic regions generally suppress behavioral action. Notably, the basolateral amygdala and medial prefrontal cortex in this context also act as a sort of arbitrator since they can drive either action or inaction depending on their projection targets. BLA, basolateral amygdala; CeA, central amygdala; dHPC, dorsal hippocampus; IfL, infralimbic prefrontal cortex; LHA, lateral hypothalamus; LS, lateral septum; NAc, nucleus accumbens; PrL, prelimbic prefrontal cortex; PFC, prefrontal cortex; OFC, orbitofrontal cortex; vHPC, ventral hippocampus.

Studies that involve vHPC lesions broadly support Gray's theory of the HPC as a behavioral inhibitor, but more recent work that has dissected the role of specific vHPC circuits, subfields, and neuron subtypes has been somewhat contradictory. While some vHPC circuits seem to block approach-related behavior, others tend to promote it (Moscarello and Maren, 2018). This new evidence suggests that the vHPC may act as a context-dependent comparator of inputs and that individual vHPC outputs function to either promote or inhibit behavioral action (Figure 3). Thus, differences in the activity of distinct vHPC circuits may drive an overreliance on approach or avoidance strategies as is found in many neuropsychiatric illnesses (Ferrante et al., 2019; Loijen et al., 2020). This review will collate recent and classical evidence related to vHPC function and present a novel role for vHPC circuit activity in arbitrating behavior under conflict, which may have implications for understanding and treating neuropsychiatric disease.

## Models of Approach-Avoidance Conflict

**Figure 4:**
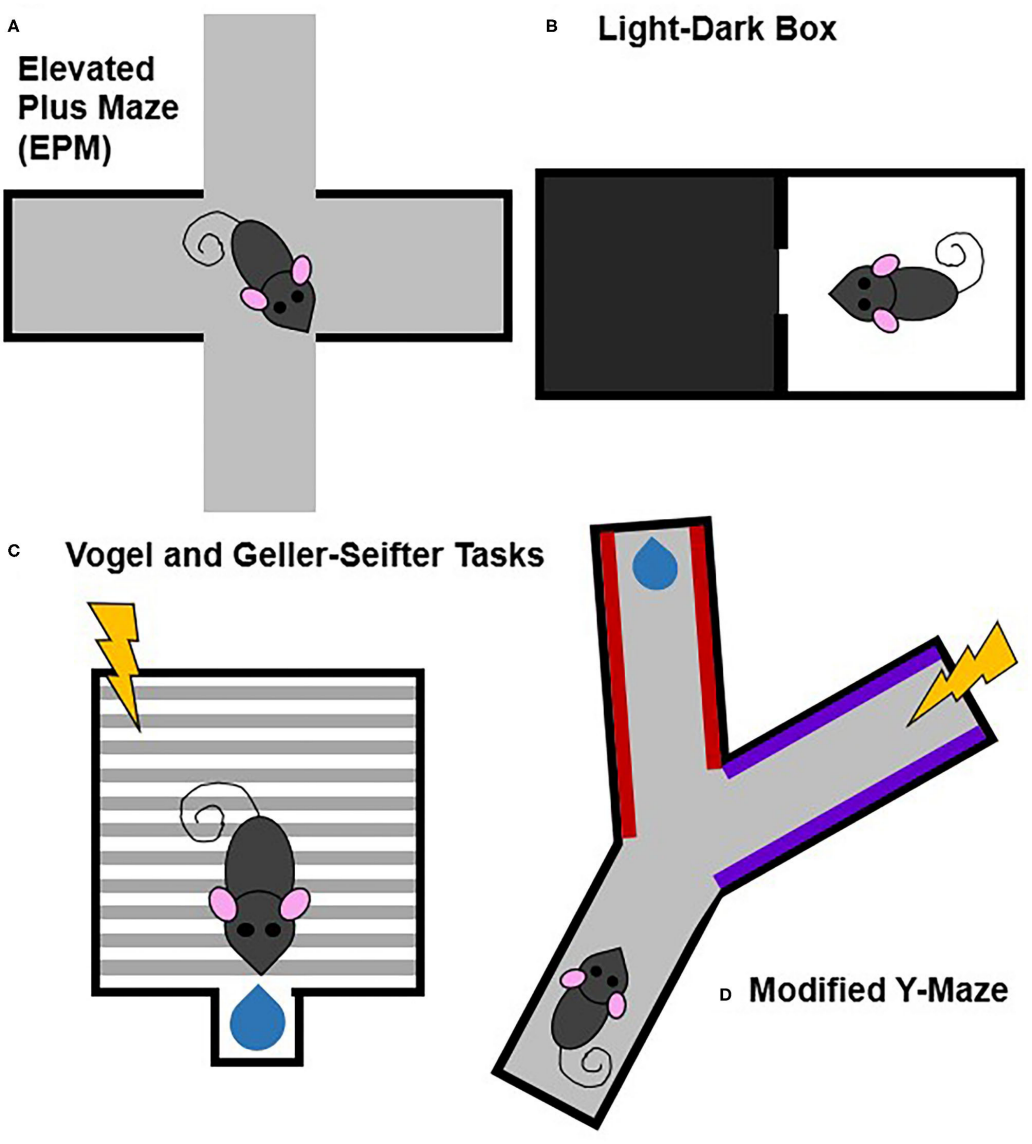
Commonly used animal models of approach-avoidance conflict. Approach-avoidance conflict tasks typically have either general or discrete cues and contexts that are associated with positive or negative outcomes. Tasks that lack stimuli associated with specifically positive or negative outcomes include the elevated plus maze **(A)** and light-dark box **(B)**. Tasks that involve discrete stimuli associated with specifically positive or negative outcomes includes the Vogel and Geller-Seifter **(C)** tasks and the modified Y-maze **(D)**.

Many animal models, especially in rodents, measure some aspect of behavior under approach-avoidance conflict (Kumar et al., 2013; Kirlic et al., 2017). Generally, these models measure conflict either through more intrinsic processes that lack discrete stimuli (e.g., drive to explore new environments vs. potential danger in a new environment) or through discrete reward- and punishment-associated stimuli (e.g., a behavior is associated with both rewarding outcomes, such as food or drug delivery, and aversive outcomes, such as footshock) (Figure 4).

Tasks that measure approach-avoidance conflict through more naturalistic, internally generated processes that lack discrete external stimuli include the elevated plus maze (EPM), the open field test, and the light-dark box (Bourin and Hascoët, 2003; Prut and Belzung, 2003; Carobrez and Bertoglio, 2005; Treit et al., 2010; Kumar et al., 2013). The conflict in these paradigms is between the drive to explore novel environments and the drive to avoid potential dangers. While these tests may be more ethological and potential contributions of differences in locomotor activity can be partially controlled for by performing appropriate analyses and comparisons, repeat testing is not ideal because of habituation to the task.

Other animal models of approach-avoidance conflict use discrete, experimenter-controlled stimuli to signal punishment and/or reward. Punishment-induced conflict tasks include the Vogel and Geller-Seifter tasks, the modified Y-maze, conditioned suppression tests, and modified conditioned place preference (CPP) paradigms (Millan, 2003; Millan and Brocco, 2003; Ito and Lee, 2016; Kirlic et al., 2017; Xie et al., 2019). Unlike the paradigms which are based off of intrinsic processes, these tasks have at least one cue or context associated with an aversive outcome (e.g., shock). Some paradigms, like the Vogel and Geller-Seifter tasks, pair shock directly with reward delivery. Other paradigms may pair shock with a cue, context, or action (e.g., lever press) that is also paired with reward. These paradigms benefit from having distinct conflicting stimuli related to explicit reward and/or punishment that is lacking in the exploratory tasks. A downfall of these approaches though is that the aversive experience likely recruits fear-related circuitry making it more difficult to tease apart approach-avoidance from fear.

These different models of approach-avoidance conflict all have important considerations as tools to study vHPC function. Even though the vHPC is not the HPC subregion most attributed to spatial navigation it also has a known role in spatial learning, particularly in respect to learning environmental contexts (Zhang et al., 2001; Ferbinteanu et al., 2003; Rudy and Matus-Amat, 2005; Hunsaker et al., 2008). Tasks that are more exploratory in nature, then, may confound the role of the vHPC in learning spatial contexts with its role in comparing conflicting stimuli. Additionally, it is also important to consider how tasks that involve active or passive avoidance may be differentially impacted by vHPC disruption. According to Gray's theory, in an approach-avoidance conflict scenario a functioning vHPC would inhibit behavior and approach by increasing the salience of negatively valenced stimuli in order to promote survival in a potentially dangerous environment (McNaughton and Gray, 2000). Yet if an action or response is required in order to avoid an aversive outcome, blocking of this response by vHPC would be detrimental. Furthermore, when the best strategy may involve instrumental action, suppression of vHPC may be required so that it does not promote innate reactions when action is required (Moscarello and Maren, 2018; Yoshida et al., 2019). In this sense, different circuits must regulate active vs. passive avoidance such that vHPC is less important for active avoidance than it is for passive avoidance, or vHPC effects on behavioral outputs are more complex than what was originally postulated by Gray.

## vHPC as an Arbitrator of Conflicting Stimuli

### Aversive Cues and Contexts

From lesion work it is clear that animals in which vHPC has been ablated seem to disregard fear-associated cues whether they are new or previously learned (Klüver and Bucy, 1937; Koh et al., 2009). The vHPC has robust bidirectional glutamatergic communication with the amygdala. Recent research suggests that vHPC projections to the amygdala, a limbic region that regulates fear response and emotional contexts (Beyeler et al., 2018), are particularly important for response to fear cues (Jimenez et al., 2018). The glutamatergic projections from vCA1 to basolateral amygdala (BLA) encode conditioned fear memory whereas glutamatergic projections to the central amygdala (CeA) are necessary for the reinstatement of a cued fear response (e.g., freezing) following extinction (Xu et al., 2016; Jimenez et al., 2018). On the other hand, the regulation of conditioned fear extinction and renewal by vHPC seems to be via its glutamatergic projections to the infralimbic (IfL) and prelimbic (PrL) prefrontal cortices, respectively (Sierra-Mercado et al., 2011; Soler-Cedeño et al., 2019; Vasquez et al., 2019). Furthermore, there is a population of neurons within vCA1 that project monosynaptically to both the medial PFC and BLA that have been found to be preferentially activated during fear renewal (Jin and Maren, 2015) and are suspected to be important in conditioned fear extinction (Ishikawa and Nakamura, 2006; Kim and Cho, 2017). Thus, the vHPC role in integrating fear-associated stimuli is at least in part through its projections to the PFC and amygdala. Furthermore, how intra-vHPC signaling impacts fear and aversion related behavior is still relatively unknown and distinct roles for vDG to vCA3 to vCA1 signaling in promoting fear response are just now being discovered (Besnard et al., 2020; Yeates et al., 2020).

It has been proposed that vHPC projections to the prefrontal cortex (PFC) act as a fear gating mechanism that determines whether reactions (e.g., freezing, fleeing) or actions (e.g., avoidance, exploration) are made (O'Donnell and Grace, 1995; Moscarello and Maren, 2018). When threat is low, PFC activation by vHPC promotes action through downstream effects on the nucleus accumbens (NAc); when threat is high, BLA activation by vHPC drives reactionary behaviors by promoting CeA activity. The vHPC to PFC projections may play similar gating roles in anxiety-like behavior. Indeed, it has been shown that inhibition of vHPC terminals within the medial PFC (mPFC) decreases anxiety-like behavior in the EPM as measured by head dips into the open arm and length of open arm visits, suggesting that this circuit normally functions to drive avoidance-like behavior (Padilla-Coreano et al., 2016). Further, increased synchrony between vHPC and mPFC oscillations has been observed in anxiogenic contexts (Adhikari et al., 2011). These findings may be contrary to the idea that vHPC to mPFC signaling promotes approach behavior. This may alternatively suggest a role for this circuit in promoting actions in the context of fear and anxiety, which may arise as either avoidance or exploration. Furthermore, the ability of the mPFC to promote approach behavior may depend on its activation of downstream targets like the NAc, and these downstream targets may differ in fear, anxiety, and reward. Thus, these findings and the extent of inter-connectivity between the vHPC, BLA, and PFC, suggest that these regions play an important role in arbitrating behavioral response to fear and anxiety.

This theory of the vHPC as a fear-gating structure is in line with the proposed theory of the vHPC as a context-dependent regulator of behavior in response to conflicting stimuli. The vHPC gathers information related to environmental cues, contexts, and emotional states and decides what strategy is most appropriate. When conflict or threat is presented some vHPC projection targets, like LHA and CeA, promote avoidance or more passive behavioral strategies while others, like BLA and PFC, promote more complex action (Figure 3). When vHPC is completely ablated, the proverbial gate is left wide open and behaviors inappropriate to the context are performed by unruly downstream targets without vHPC direction or supervision.

### Reward-Related Cues and Contexts

Compared to our understanding of vHPC contribution to fear and anxiety, its role in reward is still severely understudied. The HPC contains distinct populations of reward coding neurons that activate when seeking and tracking rewarding goals (Gauthier and Tank, 2018). Further, vHPC sends robust excitatory, glutamatergic innervation to the NAc, a region well-studied in reward (Britt et al., 2012) and vHPC inactivation has been shown to impact reward discrimination tasks (Riaz et al., 2017). This suggests that vHPC is a potent regulator of reinforcement learning and behavior. Indeed, studies have shown that vHPC input to the NAc is preferentially enhanced by dopamine D1 receptor modulation, even above amygdalar input to the NAc, suggesting vHPC has a dominant role in driving NAc activity (Charara and Grace, 2003; French and Totterdell, 2003). Beyond connectivity, it has been shown that nucleus accumbens shell (NAcS) projecting vCA1 neurons are important for the expression of sucrose-seeking habits and for the acquisition of appetitive conditioned place preference in mice and rats, respectively (Ito et al., 2008; Barker et al., 2019). This evidence suggests that the projection from vHPC to the NAc is important in maintaining motivated behavior.

As with fear, there are some projection-target specific differences in vHPC contribution to reward seeking that support a potential context-dependent role for vHPC projection targets. In the context of behavioral flexibility, inactivation of the glutamatergic vCA1 projections to the NAcS restores goal-directed sucrose seeking in mice trained to respond habitually, suggesting that the vHPC is important for the expression of habits (Barker et al., 2019). Similarly, one study showed that vHPC inactivation lead to increased ethanol drinking in non-dependent mice suggesting that vHPC may normally suppress goal-oriented drug taking (Griffin et al., 2019). However, when vCA1 neurons that project to the lateral orbital frontal cortex (OFC), a region that contains abstract representations of reward associations (Wallis, 2007), are inactivated mice defer to habitual response strategies suggesting that the vHPC is important for the expression of goal-directed behavior (Barfield and Gourley, 2019). Notably, vHPC inactivation did not impact basal reward seeking behavior in either study. If vHPC was merely an inhibitor of behavior, as proposed by the behavioral inhibition theory, one would expect goal-directed action to be suppressed by vHPC in order to divert attention to other survival processes. The fact that vHPC does not necessarily suppress goal-directed action suggests that vHPC's role in driving behavior is circuit dependent. Thus, vHPC can drive motivated behavior, but differences in behavior based on projection target support the notion that these effects are context and circuit specific (Figure 3).

### Approach-Avoidance Conflict

Approach-avoidance conflict involves the evaluation of potential rewards or punishments resulting from an action (or lack of action) as well as the likelihood of these desirable or undesirable events based on available contextual information (McNaughton and Gray, 2000; Elliot, 2006). The vHPC has a well-documented role in regulating approach-avoidance conflict (Ito and Lee, 2016), but the precise computations it performs in that role are still unknown. Studies in rodents have shown that excitotoxic lesions of vHPC reduce aversion to the open arm of an elevated plus maze and reduces secretion of stress-related hormones after exposure to a brightly lit chamber without impacting spatial or contextual memory (Kjelstrup et al., 2002; Zarrindast et al., 2008). Additionally, vHPC lesioned rats exhibit greater attention to “conflict” stimuli that are associated with both appetitive and aversive outcomes (Schumacher et al., 2016), but this also extends to “safety” stimuli that signal once an aversive outcome has been successfully avoided (Çavdaroglu et al., 2020). The vHPC may monitor all positively and negatively valenced stimuli but only drive behavior in situations where the valences overlap. In this way the vHPC acts as an arbitrator when conflict arises, and always errs on the side of caution.

One caveat of some of these studies, though, is that they involve massive vHPC lesioning. Thus, it is difficult to determine whether vHPC damage is impacting the response to or recognition of the conflicting/threatening stimuli or whether vHPC contributions are time-dependent. There is some evidence that vHPC lesioning affects the recognition of fear-associated cues, which may impact the interpretation of vHPC lesion data, but to our knowledge this has only been demonstrated experimentally with shock-associated cues thus far (Koh et al., 2009). The evidence for vHPC importance in approach-avoidance conflict is also supported by clinical research (O'Neil et al., 2015), which has shown that increasing threat levels engage the anterior HPC (human homolog of vHPC) and patients with damage to this region exhibit reduced passive avoidance (Bach et al., 2014).

Relatively recent advances in circuit manipulation methods has allowed for more complex questions regarding the precise vHPC projections that specifically regulate approach-avoidance conflict. One study showed that distinct subfields of the vHPC differentially regulate approach and avoidance such that inactivation of vCA1 induced avoidance while inactivation of vDG or vCA3 increased approach (Schumacher et al., 2018; Yeates et al., 2020). However, using fiber photometry and optogenetics Jimenez et al. (2018) found that the vCA1 region is enriched with cells that respond to anxiety-related contexts and that the activation of vCA1 to lateral hypothalamus (LHA) projecting neurons induces avoidance behavior when in anxiety-associated contexts. Notably, the neurons projecting from vCA1 to the BLA do not impact anxiety or avoidance. These findings suggest that while general inactivation of vCA1 may induce avoidance, inactivation of individual circuit outputs from vCA1 may drive approach.

Taken together, these data support the notion that vHPC is important for regulating approach-avoidance conflict, but exactly how the individual subfields are important is unclear. General vHPC ablation seems to induce approach behavior, but inactivation of specific vHPC subfields has contrasting effects, and these effects are themselves different from the effects of individual circuit manipulation. One possibility is that vHPC compares conflicting stimuli and activates different circuits depending on whether action or inaction is warranted. When large portions of the vHPC or its subfields are inactivated, the system reverts to either straight avoidance or approach as the subtlety of differences in individual computations is lost and downstream targets lose vHPC input and direction. Another possibility is that different levels of specific vHPC manipulations are affecting the balance of vHPC outputs differently. The vHPC, in particular vCA1, has externally projecting neurons that collateralize to up to 2 or 3 different regions (Gergues et al., 2020), so depending on the combination of collateralized neurons that end up get manipulated, the “weight” of vHPC input to other important downstream targets not currently being investigated may change from experiment to experiment and produce different behavioral results.

The vHPC is clearly involved in regulating conflicting stimuli but there are several potential theories related to its actual function in this process. One potential explanation is that vHPC is not involved in any sort of arbitration and just passes on information about cues in relation to the current context to downstream targets. On the other end of the spectrum, perhaps vHPC is entirely involved in comparing the stimuli and deciding on the best course of action, which it enacts through downstream targets. Finally, it is possible that the vHPC participates in both arbitration and information relay, such that arbitration of conflicting stimuli by vHPC is necessary to successfully navigate potentially dangerous scenarios but is not sufficient on its own to choose the most appropriate response.

## Discussion: Overlap and Convergence in Anxiety, Fear, and Reward Circuitry

Most of the overlap in anxiety, fear, and reward circuitry that exists in vHPC is in its projections to other major limbic structures like the PFC, NAc, and BLA. vHPC innervation to PFC is important for gating decision-making related to salient and conflicting stimuli, whether its fear- or reward-related (Yoshida et al., 2019). In particular, the PFC likely uses vHPC guidance to preferentially enact action-based strategies through downstream signaling in the NAc (O'Donnell and Grace, 1995; Moscarello and Maren, 2018), though in some contexts activation of the PFC by vHPC may also promote avoidance (Padilla-Coreano et al., 2016). Direct innervation of the NAc by the vHPC, however, suggests that signaling through the PFC is not necessary for vHPC to promote action-based strategies (Britt et al., 2012) though the types of action-based strategies enacted through direct or indirect NAc connectivity may differ (Barfield and Gourley, 2019; Barker et al., 2019). Lastly, vHPC projections to the BLA can either promote or suppress action depending on its downstream targets like the NAc and CeA, respectively (Moscarello and Maren, 2018). One interesting aspect of the PFC and BLA targets, specifically, is that a substantial portion of vHPC neurons project to both regions and have been found to be important in encoding fear contexts (Ishikawa and Nakamura, 2006; Jin and Maren, 2015). The role of these vHPC projections to the BLA and PFC in reward are still unknown but should be studied as both projection targets have been found to be important for reward and drug seeking (Kalivas, 2009; Beyeler et al., 2018). Furthermore, the vHPC also receives projections from the PFC and BLA (Beyeler et al., 2018), so how the constant conversation between these regions guides their signaling and impacts downstream targets is an area for future research. Additionally, the exact physiological mechanisms underlying vHPC regulation of approach-avoidance conflict remain generally understudied. While there exist some reports of altered vHPC physiology and synchrony in preclinical models associated with anxiety or substance use disorders (Adhikari et al., 2011; Ewin et al., 2019; Griffin et al., 2019), more research is needed in order to fully characterize vHPC function in these behaviors.

The vHPC projections to the LHA (Jimenez et al., 2018) and OFC (Barfield and Gourley, 2019) have thus far only been investigated in the context of either fear or reward, respectively. Based on the context-dependent arbitrator theory of vHPC function proposed in this review, these projection targets should regulate the same types of responses whether they are reward or fear related. Both the LHA (Jennings et al., 2013, 2015; Mangieri et al., 2018) and OFC (Milad and Rauch, 2007; Wallis, 2007) have known roles in reward and fear as well even though the role of vHPC input in regulating both of these aspects has not been researched. Further support for this theory comes from work looking at the role of the vHPC projection to the lateral septum (LS) in both reward and fear. A very recent study has shown that vCA3 projections to the dorsal LS suppress fear response (Besnard et al., 2020), similar to the function of vHPC projections to the LS in suppressing feeding (Sweeney and Yang, 2015). This is perhaps not too surprising as the HPC to septum projections (labeled as the Septal Hippocampus System or SHS) were central to Gray's theory of the behavioral inhibition system. Still, as would be suggested by this theory, the vHPC projections support only one type of response (in the case of the LS, to suppress behavioral action) regardless of whether it is fear or reward related.

Together, current evidence indicates that theories of vHPC contribution to behavioral inhibition should be updated. vHPC is not entirely a suppressor of action, and instead is a context-dependent decider of behavioral strategy. Depending on whether an action in a particular context should be suppressed or promoted, vHPC signals to different regions to this effect. When vHPC is severely damaged or ablated, there is no longer a decider present to promote or suppress certain behaviors, so actions that are normally suppressed in a particular context are performed unabated.

## Author Contributions

KB: research, writing, and figures. KB and JB: editing and theorizing. Both authors contributed to the article and approved the submitted version.

## Conflict of Interest

The authors declare that the research was conducted in the absence of any commercial or financial relationships that could be construed as a potential conflict of interest.
